# Interspecies recombination in NSP3 gene in the first porcine rotavirus H in Russia identified using nanopore-based metagenomic sequencing

**DOI:** 10.3389/fvets.2023.1302531

**Published:** 2023-12-05

**Authors:** Nikita Krasnikov, Anton Yuzhakov

**Affiliations:** Laboratory of Biochemistry and Molecular Biology, Federal State Budget Scientific Institution “Federal Scientific Center VIEV” (FSC VIEV), Moscow, Russia

**Keywords:** rotavirus H, swine diseases, nanopore sequencing, phylogenetics, metagenomics, viral recombination

## Abstract

During the last decade, porcine rotavirus H was detected in the USA, Asian regions, South Africa, Brazil, and a couple of European countries. In the presented study, the virus was identified in piglets on a farrow-to-finish farm in Russia during metagenomic surveillance. Currently, it is the first identification of this species in the country. As a diagnostic method, nanopore-based metagenomic sequencing was applied. The obtained nanopore reads allowed for the assembly of 10 genome segments out of 11. The phylogenetic analysis revealed the virus belonged to the porcine cluster and had GX-P3-I3-R3-C3-M8-A7-N1-T5-E3-H3 genome constellation. Moreover, three potential new genotype groups for VP3, NSP1, and NSP3 genes were determined. Additionally, a recombination between RVH and RVC in the NSP3 gene was detected. The study provides significant information about a novel RVH strain.

## Introduction

Rotaviruses (RVs) are the most common cause of diarrhea in humans and animals, including pigs (1). The rotavirus genome is represented by linear double-stranded RNA (dsRNA) and consists of 11 segments that encode six structural proteins (VP1–VP4, VP6, and VP7) and five to six nonstructural proteins (NSP1–NSP5/6). RVs belong to the *Sedoreoviridae* family and are currently classified into nine species, A to J, based on the sequence diversity of the inner capsid protein (VP6) (2).

RV infections are quite common on swine farms and frequently associated with post-weaning and suckling diarrhea, which cause significant financial losses for the pork industry. Among the nine RV species, RVA, RVB, RVC, and RVH have been identified in fecal samples from pigs of different ages. Besides, Rotavirus E stands out, but it was revealed only once in 1986 and has not been studied genetically (3), so it has been excluded from the RV classification.

RVA, RVB, and RVC are widely spread in swine herds worldwide (1). RVA mostly affects piglets between 3 and 6 weeks of age (4, 5). RVB infections are generally associated with pigs >3 weeks old and occasionally cause diarrhea in neonatal piglets (5), while RVC is more commonly seen in young piglets (< 7 days of age) and is often associated with neonatal diarrhea (5, 6). At the same time, there is still no evidence of RVH pathogenic influence on pig health.

There is limited information on the geographical distribution and genetic characterization of RVH. The virus was originally identified in humans with symptoms of gastroenteritis in China in 1997 and named “adult diarrhea rotavirus” (ADRV-N) (7). Afterward, a similar virus was detected in Bangladesh in 2002 (the B219 strain) (8). These strains were genetically related to group B rotaviruses to some extent and close to them ecologically, but then were separated into a distinct branch based on VP6 sequence analysis (9, 10).

Subsequently, the RVH circulation was confirmed in pigs of different ages in the USA (11, 12), Japan (13, 14), Brazil (15, 16), South Africa (17), Vietnam (18), and some European countries, such as Spain (19) and Italy (20). A recent large-scale study in China has also indicated a high prevalence of RVH in the local swine herds (21). Furthermore, the virus has also been observed in bats in west-central Africa (22) and Korea (23). There were also some studies that demonstrated detection in bat metagenomes (24, 25). In this study, we characterize the first identified isolate of porcine RVH in Russia and discuss its phylogenetic relationships with known RVH strains.

## Materials and methods

### Sample collection

During metagenomic surveillance in the fall of 2022, within the framework of the project “TechPEPCon,” we collected nasal and rectal swabs from three piglets on the 10th week of life. Finally, three distinct samples of one type from three different piglets were pooled into one. The case farm was located in the Moscow region of Russia and positioned as healthy. According to the herd veterinarian, the animals had no clinical manifestations of infections. For taking swabs and further sample filtering, a specially designed sampler was used (PathoSense BV, Belgium). The samples were transported in Dulbecco's phosphate-buffered saline (dPBS) at 4°C to the lab for immediate subsequent analysis.

### Nanopore sequencing

The lab diagnostic procedure was performed according to a protocol developed by PathoSense BV (Merelbeke, Belgium). Briefly, it included nucleic acid extraction using the Quick-DNA/RNA viral kit (Zymo Research, CA, USA), reverse transcription using the SuperScript IV Reverse Transcriptase (ThermoFisher Scientific, USA) with following enrichment by PCR (KAPA HiFi HotStart ReadyMix; Roche, Switzerland). Then amplicons were cleaned with the magnetic beads (AMPure XP; Beckman Coulter, USA) in a ratio 1:1. Final quantity and quality were verified using NanoDrop OneC spectrophotometer (ThermoFisher Scientific, USA). For the library preparation, the Rapid Barcoding Kit (RBK-004; ONT, Oxford, UK) was used. After that, nanopore sequencing was performed on a MinION flow cell (R9.4.1; ONT, Oxford, UK) for 6 h.

### Metagenomic analysis and genome assembly

FASTQ files were generated and demultiplexed using Guppy basecaller (v. 21.10.4) in the super-accurate basecalling setting. Quality filtering was performed using NanoFilt (v. 2.6.0) (26). Reads with a q-score <7 were omitted. Swine host reads were removed after alignment to the Sus scrofa genome (GCA_000003025.6) using graphmap (v. 0.5.2) (27) and samtools (v. 1.6) (28). For metagenomic analysis, filtered sequences were scanned using the BLASTn method (BLAST v. 2.13.0) with customized nucleotide databases. The hits with lowest e-value were visualized using KronaTools (v. 2.8.1) (29).

The nanopore segment assembly was performed as follows: minimap2 (v. 2.24) (30) was used for mapping filtered reads according to their genome segment, and samtools (v. 1.6) (28) was used to generate an individual segment FASTQ file. For each segment, the corresponding FASTQ file was taken to perform de novo segment assembly using Canu (v. 2.2) (31), minimap2 (v. 2.24) (30), and several rounds of medaka polishing (v. 1.7.2; ONT) for generating final consensus sequences. The sequence coverage was different between the segments from 20× to 150× (Supplementary Table 1). The quality of the assembled segments was assessed by comparison with publicly available sequences in NCBI GenBank.

### Phylogenetic and recombination analyzes

The obtained sequences were aligned by the ClustalW algorithm. Genetic distances were calculated using the Kimura two-parameter correction at the nucleotide level. Phylogenetic dendrograms were plotted using the maximum likelihood (ML) method and the GTR (G+I) model (MEGA 7.0) (32). The robustness of the topology was evaluated by 1000 bootstrap replications. The visualization of the Newick files of phylogenetic dendrograms was performed using iTOL v6 (33). Recombination events were evaluated using the Recombination Detection Program (RDP) (v. 4.101) with RDP, GENECOV, Bootscan, MaxChi, Chimera, SiSscan, and 3Seq algorithms (34). Similarity plot analysis was conducted using the SimPlot software (v. 3.5.1) with a sliding window of 200 bp (step: 20 bp).

## Results

### Metagenomic nanopore-based analysis

In the presented study, RVH (isolate KLM-22) was detected in rectal samples of ten-week-old piglets while conducting nanopore-based metagenomic surveillance in the Moscow region of Russia. The rectal sample was composed of three distinct rectal samples from three different piglets. Taking into account other swine viruses, the metagenomic composition also included porcine torovirus, porcine sapelovirus, porcine kobuvirus, porcine enterovirus 9, and porcine astrovirus 4, composed of 1–3% each. Among bacterial species, the metagenomic approach revealed *Prevotella sp., Campilobacter sp*., *Bacteriodes sp*., and *Chlamydia suis* in low quantities.

As a result of the nanopore sequencing, complete RVH segment sequences were achieved for VP2, VP3, VP6, NSP1, NSP2, NSP3, NSP4, and NSP5 and partial sequences for VP1 and VP4. There were some reads of VP7, but not enough to assemble the sequence consensus properly. All obtained sequences of the detected isolate KLM-22 were deposited in the NCBI GenBank with the accession numbers: OQ603399–OQ603403 and OR589274-OR589278.

### Phylogenetic analysis

**Figure 1:**
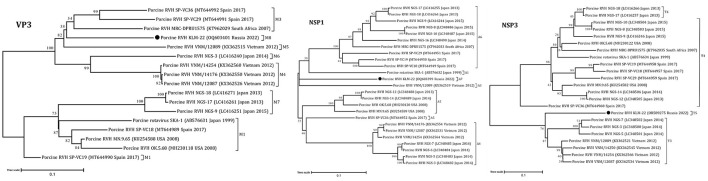
Phylogenetic dendrograms constructed with the GTR model for the VP3, NSP1, and NSP3 RVH segments. Three novel genotype groups M8, A7, and T5 were determined in this study. The filled circles above the strains indicate the RVH strain identified in this study. Scale bars indicate nucleotide substitutions per site. GenBank accession number, country, and collection year of each sample are also shown. Genotypes are specified in accordance with previous studies (13, 19) and indicated on the right of the bracket.

**Figure 2:**
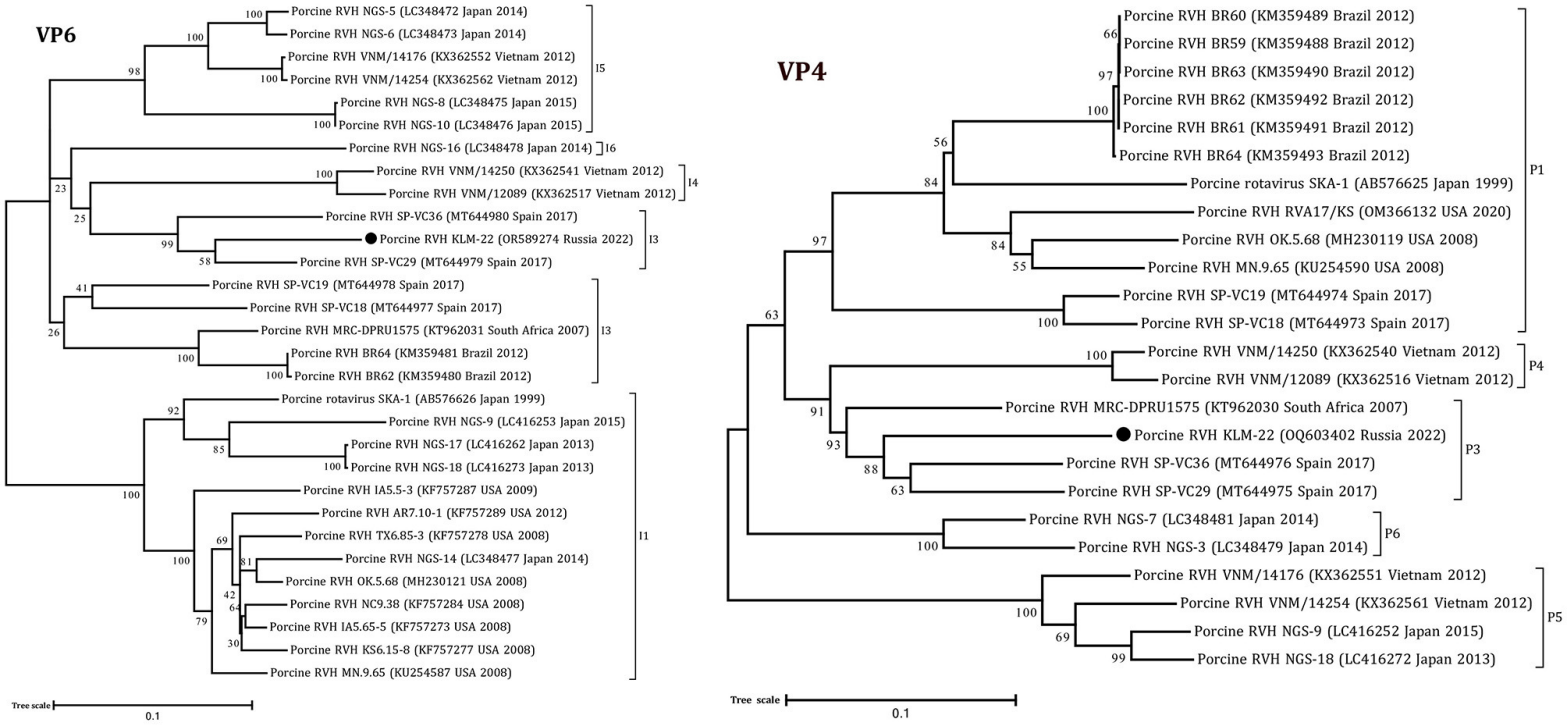
Phylogenetic dendrograms constructed with the GTR model for the VP4 and VP6 RVH segments. The filled circles above the strains indicate the RVH strain identified in this study. Scale bars indicate nucleotide substitutions per site. GenBank accession number, country, and collection year of each sample are also shown. Genotypes are specified in accordance with previous studies (13, 19) and indicated on the right of the bracket.

The obtained RVH segment sequences were used for phylogenetic analysis, including complete and partial genome sequences of RVH isolates from NCBI GenBank. According to the constructed ML trees, the sequences were related to a porcine RVH cluster (Figures 1, 2, Supplementary Figures 2–6). The pairwise identity among the presented RVH isolate and GenBank sequences varied between 71 and 86% for VP4, 78–90% for VP6, 78–86% for VP1, 82–88% for VP2, 78–85% for VP3, 74–82% for NSP1, 85–88% for NSP2, 74–83% for NSP3, 70–84% for NSP4, and 83–93% for NSP5 (Supplementary Data Sheet 2). Analysis of VP3, NSP1, and NSP3, based on cut-off values of 86, 84, and 87%, revealed the presence of three potentially new RVH genotype groups: M8, A7, and T5, respectively. Apart from that, the sequences of these segments were related to the distinct clades according the corresponding constructed phylogenetic dendrograms (Figure 1).

According to the ML trees and phylogenetic analysis of VP6 and VP4 segments, the KLM-22 RVH strain was closer to the strains from Spain and South Africa (Figure 2). Considering the ORF variability of the NSP1 segment, it was characterized as a long type and was 1206 bp in length.

Finally, taking into account the full genome-base genotyping system for RVH with cut-off nucleotide identity values, advanced by Suzuki and Inoue (13), we classified the detected strain as follows: GX-P3-I3-R3-C3-M8-A7-N1-T5-E3-H3.

### Recombination in NSP3 gene

The NSP3 segment sequence was related to a long type, as its ORF length exceeded the standard (around 801 bp) and equaled 1248 bp. To investigate the origin of the extra nucleotide sequence in long-type NSP3, a BLAST search method was performed. The 448 nucleotide sequence (position 815–1262) from the NSP3 sequence displayed a high degree of resemblance with the middle regions (positons 546–987 bp) of two porcine RVC strains from the USA: RVC/Pig-wt/USA/IA46/2012 (GenBank: MG451244.1) - 86.65%, and RVC/Pig-wt/USA/MN_1233/2013 (GenBank: MT761794.1) - 85.97%, using the BLASTn method (Supplementary Figure 7). The recombination event was additionally verified by RDP analysis with a set of the genetically closest sequences by BLASTn (Supplementary Table 2), and following tests with corresponding *p*-values proved it (Table 1).

**Table 1:** Recombination event identified using the Recombination Detection Program (RDP) v.4.101 for rotavirus H NSP3 gene.

**Strain name, accession No**.	**Gene**	**Representative major parent from GenBank**	**Representative minor parent from GenBank**	***p*** **-values**	
RVH/Pig-wt/RUS/KLM-22/2022 (OR589275)	NSP3	RVC/Pig-tc/USA/Cowden/1991/G1P1 (M69115)	RVC/Pig-wt/USA/IA46/2012/G9P7 (MG451244)	RDP	–
				GENECONV	–
				BootScan	4,470 × 10^−09^
				MaxChi	3,603 × 10^−07^
				Chimera	1,198 × 10^−07^
				SiScan	1,229 × 10^−08^
				3Seq	2,456 × 10^−02^

**Figure 3:**
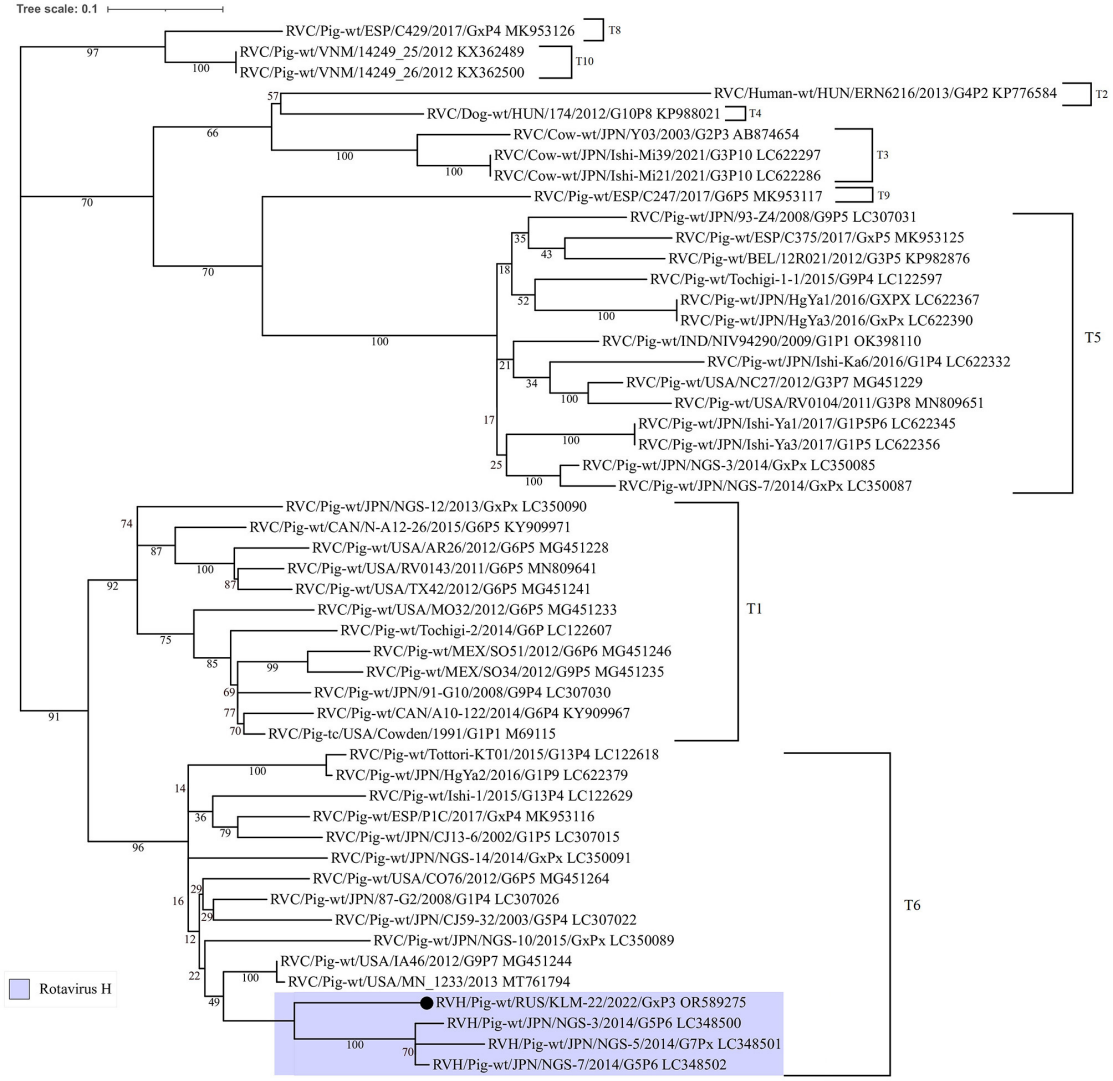
Phylogenetic dendrogram constructed from partial NSP3 open reading frame nucleotide sequences (448 bp) from RVC strains, including three porcine RVH strains from Japan and the studied one. The dendrogram was constructed using the maximum-likelihood method with MEGA 7 software and visualized using iTOL v6. The number near each node displays the percentage of bootstrap support (for 1000 replicates) for the cluster. Genotypes are indicated on the right of the bracket.

The similarity plot analysis also showed a high identity level with RVC strains in the alignment interval from 800 to 1250 bp (Supplementary Figure 1). Additionally, to understand the phylogenetic relationship of the recombinant site from RVC, the corresponding dendrogram was constructed. For comparison, partial NSP3 nucleotide sequences from different rotavirus C strains, including insertions from three Japanese RVH strains (LC348500, LC348501, and LC348502), were selected (Figure 3).

According to the ML tree, the RVC site was closer to the insertions from the Japanese RVH strains and related to the T6 genotype by the RVC classification system (35). Moreover, the insertion in question was longer, than Japenese in 27 bp.

## Discussion

RVH is a poorly studied species among porcine RVs. Previously, it was detected in swine herds only in eight countries worldwide. In our study, the virus was identified in piglets on the 10th week of life, which correlates with the work by Nyaga et al. (17) in South Africa. Numerous studies also indicate that the virus was more often circulated among weaning and fattening classes, in age groups of 21–70 days, than during the suckling period (12, 19, 20).

RVs, including RVH, can be detected in pigs with and without diarrhea (15). According to the study of Marthaler et al. (12), RVH was most frequently detected in co-infection with A, B, and C species. During the large-scale epidemiological study in Italy, RVH was also found mostly in combinations with other RVs and was predominant in only 0.9% of the samples among single RV infections (20). The recent monitoring study in China demonstrated a high positivity rate for single RVH cases in the clinical samples: 13.68% (190/1447). Otherwise, in co-infections, RVH was noted more rarely: RVA+RVH (2.14%), RVB+RVH (0.83%), and RVC+RVH (1.66%). It is also interesting that the RVA + RVB + RVC + RVH infection showed a positivity rate of 2.07% (30/1447) (21).

The studies likewise specify that the virus is more of an opportunistic agent than a severe pathogen (12, 36). Despite the fact that RVH was occasionally detected in pigs with diarrhea, the virus role in the infection development has yet to be proven experimentally, and the question of RVH pathogenicity is still open. In our case, there were no clinical signs in the studied piglets on the 10th week, and among RV species, RVH was the only one detected. The presented observation demonstrates that RVH can freely circulate in a herd without causing any clinical signs.

The metagenomic approach, in conjunction with ONT sequencing, is a convenient pathogen diagnostic instrument (37). Regarding RVs, the method allows identifying species and subtypes and generating segment assemblies (38, 39). In the given case, a nanopore-based metagenomic approach allowed us to obtain 10 segments out of 11. The phylogenetic analysis suggested an absence of interspecies transmission between pigs, humans, and bats in the presented case. Additionally, the study expanded the current RVH genotype classification and revealed potentially new genotypes for VP3, NSP1, and NSP3.

Gene rearrangements between homologous and heterologous strains are crucial driving forces in the evolution of RVs. There are plenty of studies evidencing reassortation and recombination events among strains of the same rotavirus species (40–42). On the other hand, between different species, such genetic changes happen much less frequently. During the analysis, we identified interspecies recombination in the NSP3 gene between rotavirus H and C. A similar recombination finding has already been noted by Suzuki and Inoue (13) in Japanese RVH strains. This fact leads us to think that such genetic events can occur not accidentally but periodically between these species and deserve further investigation. It is commonly known that rotavirus NSP3 is a surrogate of the Poly(A) Binding Protein-Poly(A) Complex (PABP) (43). The protein evicts PABP from its binding site on eIF4G and binds the 3′-end of viral mRNA and cellular eIF4G, which inhibits host translation and thereby promotes viral one. Simultaneously, the complete functional role of NSP3 in porcine RVH and RVC remains unstudied, and future analyses are required.

## Conclusion

In this work, we report the genome sequences of porcine RVH, the first RVH strain identified in Russia. In our case, the virus was detected in piglets during the 10th week of life, between the nursery and fattening periods. We also present a phylogenetic analysis of the 10 segment sequences that showed their porcine RVH cluster membership. Additionally, we demonstrate the gene recombination between rotavirus H and C in NSP3.

The study also emphasizes the importance of epidemiological surveillance at later stages of growth, including the fattening period. Such a procedure can provide full insight regarding animal health and reveal previously unknown pathogens for the exact herd. It is also of great value to conduct phylogenetic and recombination studies to evaluate genetic transformations and predict the rotavirus evolution, and nanopore-based metagenomic sequencing copes with all of the above tasks.

Our data confirm that RVH presently circulates among domestic pigs in Russia. However, detailed monitoring of swine herds on farms is required to fully understand RVH diversity and the currently existing epidemiological situation.

## Data availability statement

The datasets presented in this study can be found in online repositories. The names of the repository/repositories and accession number(s) can be found in the article/Supplementary material.

## Ethics statement

The animal studies were approved by the Local Ethical and Animal Welfare Committee of the Federal State Budget Scientific Institution Federal Scientific Center VIEV (Moscow, Russia). The studies were conducted in accordance with the local legislation and institutional requirements. Written informed consent was obtained from the owners for the participation of their animals in this study.

## Author contributions

NK: Conceptualization, Data curation, Formal analysis, Investigation, Methodology, Software, Validation, Visualization, Writing – original draft. AY: Data curation, Funding acquisition, Project administration, Resources, Supervision, Writing – review & editing.
