# Supplementary material for: Interspecies recombination in NSP3 gene in the first porcine rotavirus H in Russia identified using nanopore-based metagenomic sequencing
Source: Front Vet Sci. 2023 Dec 5;10:1302531. doi: 10.3389/fvets.2023.1302531 (PMC10728476; doi:10.3389/fvets.2023.1302531)

Supplementary Material

Supplementary Table 1. Number of reads used for the assembly and final coverage for each segment of KLM-22 RVH isolate in this study.

| Segment | Segment sequence length (bp.) | Number of reads for assembly (q-score ≥7) | Coverage |
| --- | --- | --- | --- |
| VP1 | 3365 (partial) | 225 | 40× |
| VP2 | 3001 | 550 | 72× |
| VP3 | 2197 | 335 | 66× |
| VP4 | 2432 (partial) | 474 | 150× |
| VP6 | 1286 | 120 | 52× |
| NSP1 | 1323 | 249 | 50× |
| NSP2 | 1002 | 74 | 24× |
| NSP3 | 1445 | 299 | 108× |
| NSP4 | 747 | 56 | 23× |
| NSP5 | 666 | 44 | 20× |

**Supplementary Table 2.** Sequences used by the Recombination Detection Program (RDP) v. 4.101 to evaluate the recombination event in the NSP3 gene between rotaviruses C and H.

| Isolate name | GenBank accession number |
| --- | --- |
| RVC/Pig-wt/USA/IA46/2012/G9P7 | MG451244 |
| RVC/Pig-wt/USA/MN_1233/2013 | MT761794 |
| RVC/Pig-wt/JPN/87-G2/2008/G1P4 | LC307026 |
| RVC/Pig-wt/USA/CO76/2012/G6P5 | MG451264 |
| RVC/Pig-wt/JPN/CJ13-6/2002/G1P5 | LC307015 |
| RVC/Pig-wt/JPN/CJ59-32/2003/G5P4 | LC307022 |
| RVC/Pig-tc/USA/Cowden/1991/G1P1 | M69115 |
| RVC/Pig-wt/CAN/N-A12-26/2015/G6P5 | KY909971 |
| RVC/Cow-wt/JPN/Ishi-Mi39/2021/G3P10 | LC622297 |
| RVC/Cow-wt/JPN/Ishi-Mi21/2021/G3P10 | LC622286 |

**Supplementary Figure 1.** Similarity analysis of the NSP3 gene of the RVH KLM-22 isolate with complete RVC NSP3 sequences. GenBank accession numbers of RVC sequences are shown on the right of the plot. The analysis was performed by SimPlot 3.5.1 (10) using a sliding window of 200 nucleotides moving in 20-bp steps.

**
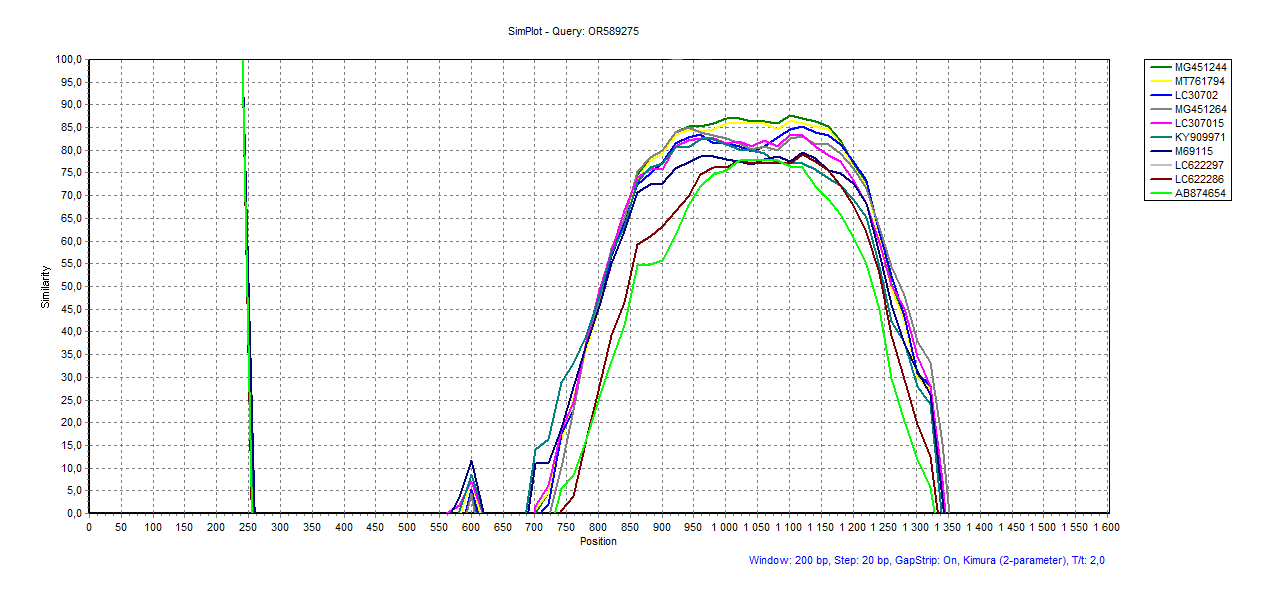
**

**Supplementary Figure 2.** Phylogenetic dendrogram constructed for the VP1 RVH segments.


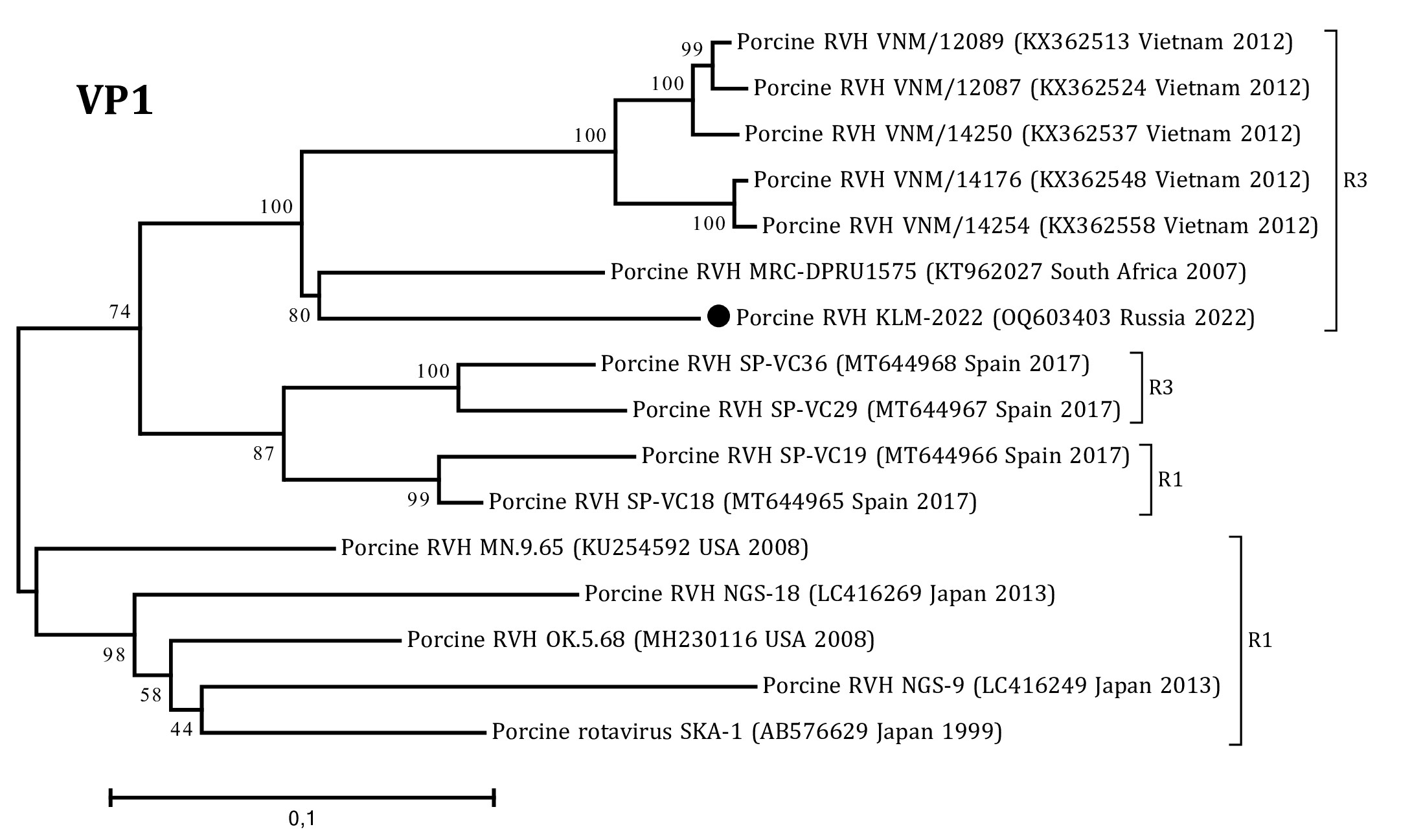


**Supplementary Figure 3.** Phylogenetic dendrogram constructed for the VP2 RVH segments.

**
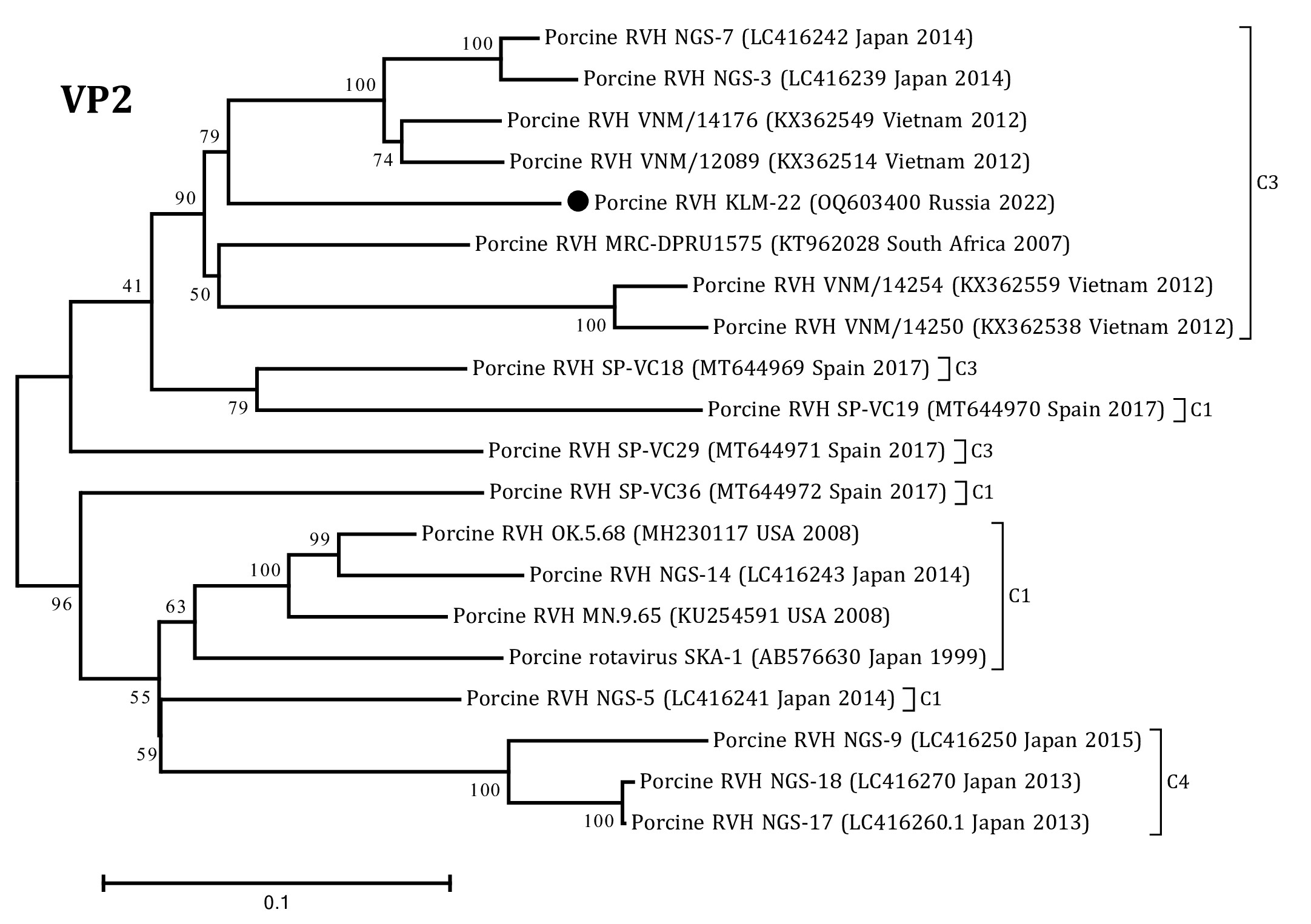
**

**Supplementary Figure 4.** Phylogenetic dendrogram constructed for the NSP2 RVH segments.

**
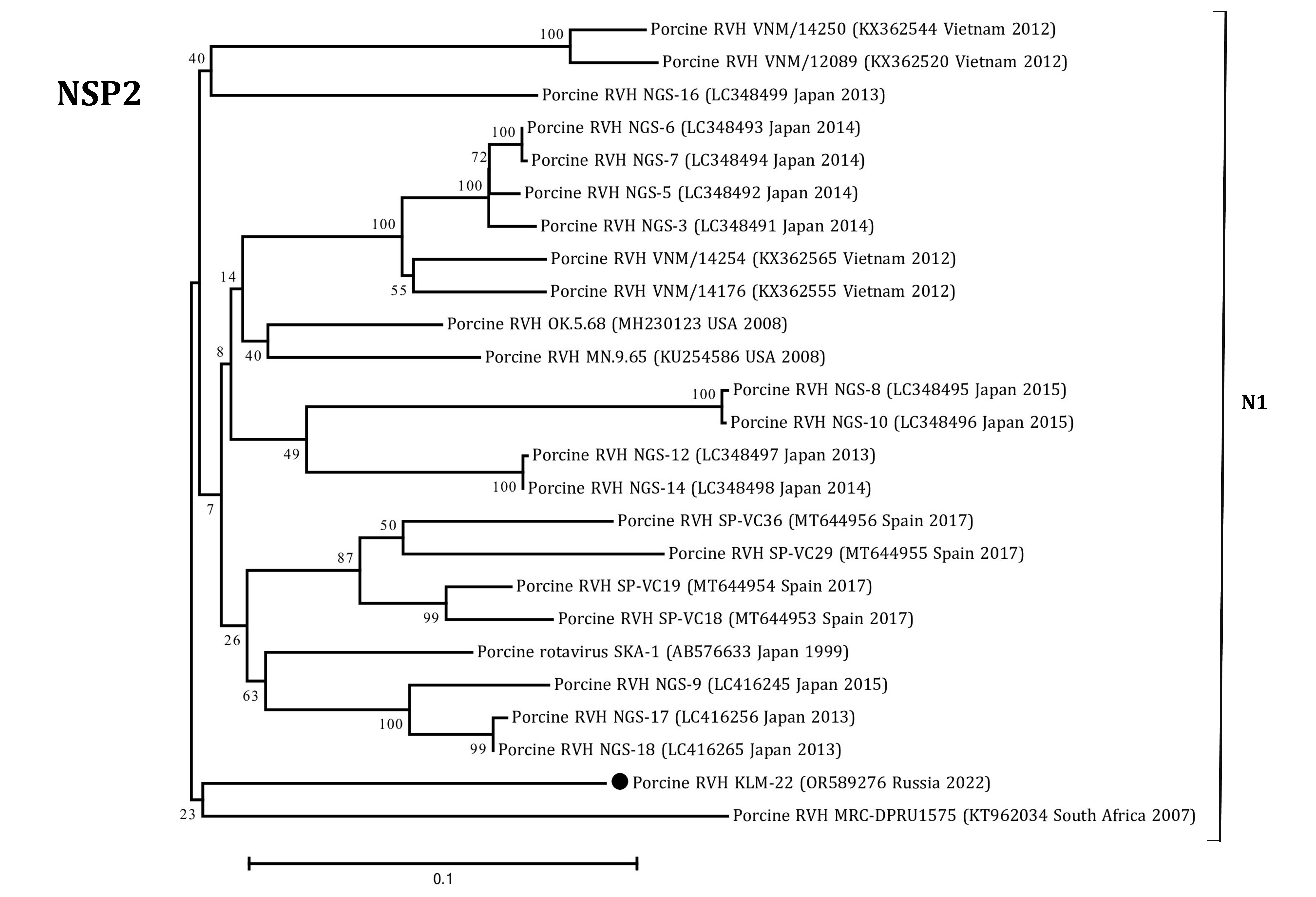
**

**Supplementary Figure 5.** Phylogenetic dendrogram constructed for the NSP4 RVH segments.


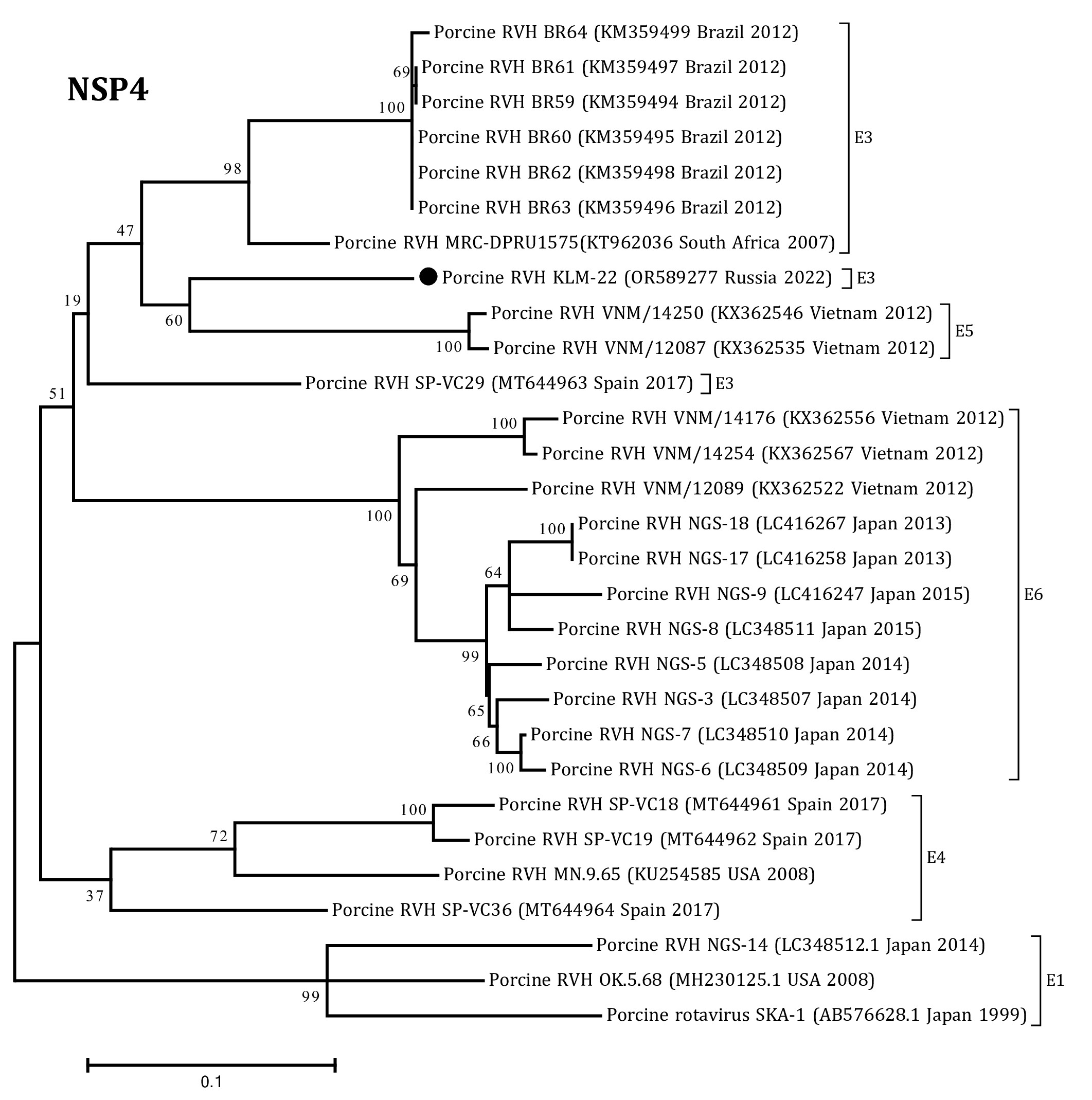


**Supplementary Figure 6.** Phylogenetic dendrogram constructed for the NSP5 RVH segments.


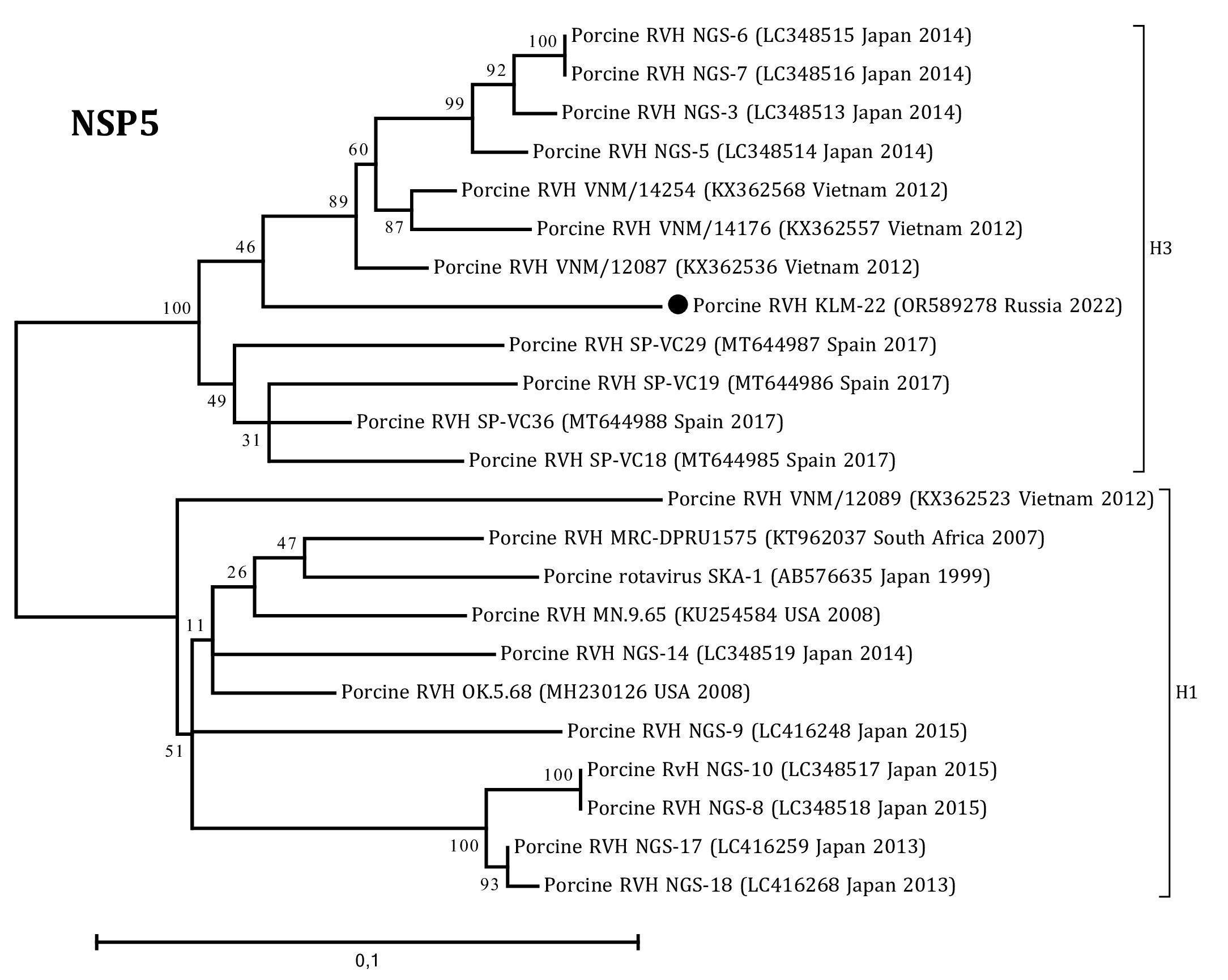


**Supplementary Figure 7.** Schematic illustration of gene recombination between porcine RVH and porcine RVC in the NSP3 gene.


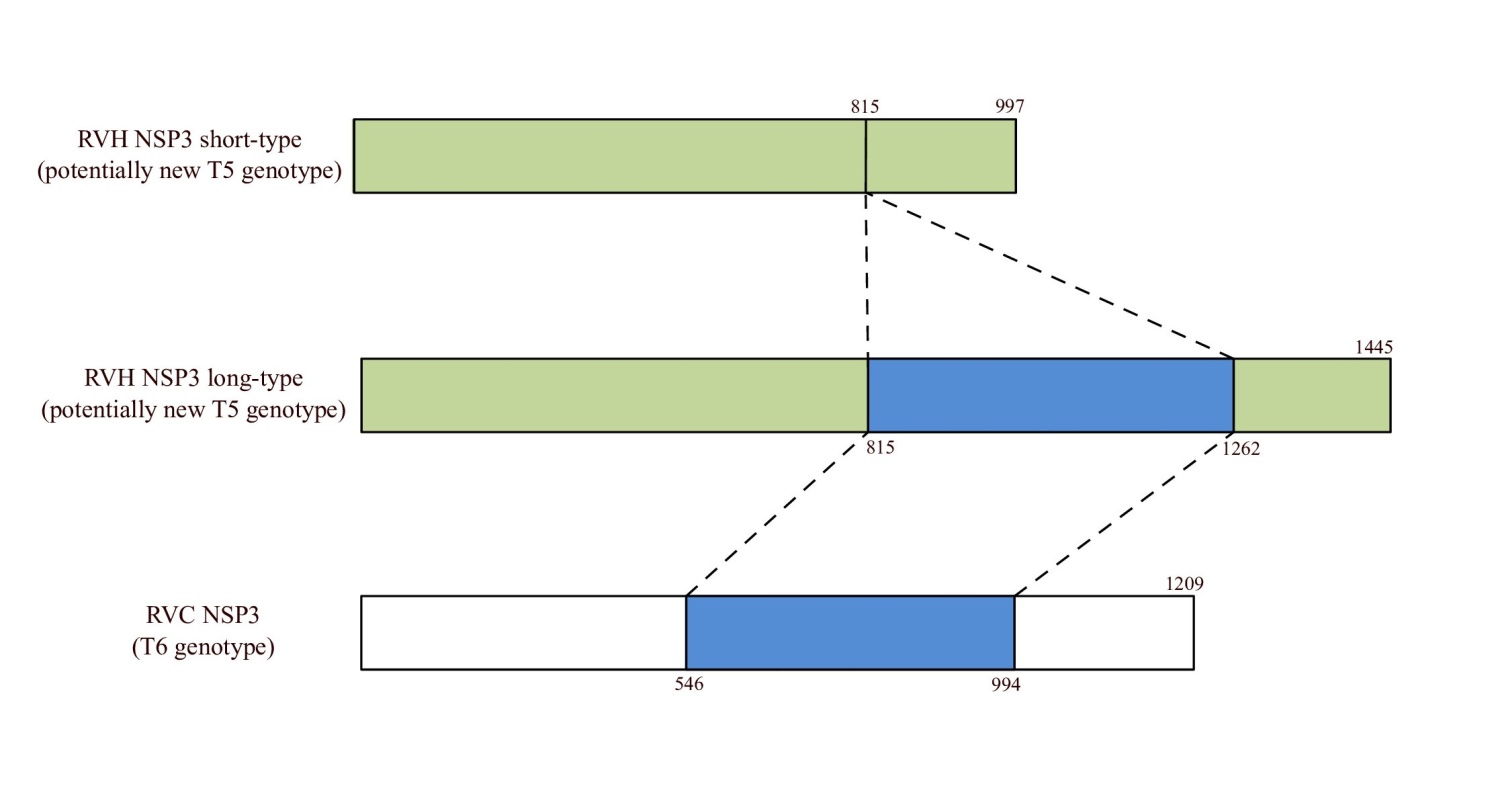

Supplement: Supplementary file 1 [file Data_Sheet_1.docx]
